# To Medical Colleges

**Published:** 1867-10

**Authors:** 


### Editorial Department.

### To Medical Colleges.

At the Convention of Delegates from Medical Colleges, called for the purpose of revising the system of Medical College instruction in this country, and which convened in Cincinnati, May 3d, 1867, the following resolution was unanimously adopted:

"*Resolved*, That a committee of five be appointed by the President, whose duty it shall be to present the several propositions adopted by this Convention, to the Trustees and Faculties of all the Medical Colleges in this country, and solicit their definite action thereon, *with a view to the early and simultaneous practical adoption* of the same throughout the country. And that the same committee be authorized to call another convention when deemed advisable."

The undersigned committee, appointed for the purpose of carrying into effect the instructions contained in the foregoing resolution, respectfully invite the attention of the Trustees and Faculty of each duly organized Medical College in the United States to the four following propositions, of great practical importance, namely:—1st. A positive standard of preliminary education. 2d. A longer time in which to acquire a knowledge of the various branches of Medical Science and practice. 3d. A systematic and successive order of studies for the student. 4th. A certain amount of direct clinical instruction in a public hospital as a part of the senior course. The desirableness of these changes is too apparent to require either argument or illustration. The plan for accomplishing them, adopted by the convention, as expressed in the foregoing propositions, is simple and easy of execution, provided the several colleges will act in concert.

It requires each college to obtain and place on record sufficient evidenee that every student admitted to matriculation possesses a certain amount of preliminary education. It requires *attendance* and *pay* for three annual courses of college instruction, as a condition for graduation; and arranges the whole curriculum of the college into three corresponding series of branches, so that each studeut can limit his attention to one ser ies each year, thereby laying a foundation and building on it a superstructure in their natural order. * * * * * We therefore respectfully ask you to give it a full consideration, and return to the Chairman of the undersigned Committee answers to the following questions:

1st. Do your Faculty, together with the governing authority of your college, approve of the several propositions as a whole?

2d. If yon do not approve of the plan of revision as a whole, what changes would you suggest?

3d. If you approve of the plan as a whole, or of all its essential features, will your college be ready to adopt it practically, and issue your Annual Announcement for the College term of 1868–9, in accordance therewith; provided all the principal Medical Colleges in this country (or at least those in the cities of Boston, New York, Philadelphia, Baltimore, Richmond, Charleston, New Orleans, Louisville, Cincinnati, St. Louis, Chicago, Buffalo and Albany,) will agree to do the same at the same time?

The great desideratum is to secure both harmony and concert of action on the part of the Medical Colleges, in the adoption of such measures as will at once place the system of medical education in this country on such a basis as the extent of the science and the responsibilities of its practical application in the prevention and treatment of diseases, require.

N. S. DAVIS, GEO. C. BLACKMAN,
S. D. GROSS, F. DONALDSON,
Chicago, Aug. 1st, 1867. Committee.

We have not published this Circular entire, for want of space.—ED.

